# Lateral-drag propulsion forces induced by anisotropy

**DOI:** 10.1038/s41598-017-06307-8

**Published:** 2017-07-21

**Authors:** Igor S. Nefedov, J. Miguel Rubi

**Affiliations:** 1 Aalto University, School of Electrical Engineering, P.O. Box 13000, 00076 Aalto, Finland; 2 Laboratory Nanooptomechanics, ITMO University, St. Petersburg, 197101 Russia; 3 Statistical and Interdisciplinary Physics Section, Departament de Fisica de la Matèria Condensada, Universitat de Barcelona, Marti i Franquès 1, 08028 Barcelona, Spain

## Abstract

We predict the existence of lateral drag forces near the flat surface of an absorbing slab made of an anisotropic material. The forces originate from the fluctuations of the electromagnetic field, when the anisotropy axis of the material forms a certain angle with the surface. In this situation, the spatial spectra of the fluctuating electromagnetic fields becomes asymmetric, different for positive and negative transverse wave vectors components. Differently from the case of van der Waals interactions in which the forward-backward symmetry is broken due to the particle movement, in our case the lateral motion results merely from the anisotropy of the slab. This new effect, of particular significance in hyperbolic materials, could be used for the manipulation of nanoparticles.

## Introduction

Fluctuating electromagnetic fields are responsible for important phenomena such as thermal emission, radiative heat transfer, van der Waals interactions, Casimir effect, and van der Waals friction between bodies^1^. The existence of attractive forces between two perfectly conducting parallel plates, induced by vacuum fluctuations at zero temperature, was predicted by Casimir in 1948^2, 3^ and subsequently by Lifshitz^4^ for any media at finite temperature. A general electromagnetic fluctuation theory, referred to as fluctuational electrodynamics, was proposed by Rytov in 1950^5^. The conventional Casimir force between two parallel surfaces is orthogonal to the surfaces, of attractive or repulsive nature depending on the separation distance and on the medium filling the gap between surfaces^6, 7^.

The lateral component of the Poynting vector, integrated over the whole spatial spectrum, vanishes near flat surfaces because its positive and negative components balance each other out. This symmetry can be broken by a mutual lateral movement of the bodies, as happens in the case of contact-free van der Waals and quantum friction^8, 9^. To observe these forces, one applies an electric current to a conducting layer and measures the friction drag effect of electrons in a second parallel metallic layer^10, 11^. Lateral drag forces can also be present nearby surface inhomogeneities, such as corrugations^12–14^. These forces, however, can only give rise to local displacements that follow the periodicity of the corrugations^15–17^ and not to a net movement of the particle over an appreciable distance. A lateral propulsion force, exerted on an anisotropic particle in a non-equilibrium regime, was predicted by Müller and Krüger^18^. In the situation analyzed, the forces acting on the particle cause not only lateral motion but also rotation leading the particle to a state of minimal energy where lateral motion ceases. Anisotropy can also induce van der Waals torques, predicted between anisotropic half-spaces^19^, anisotropic cylinders^20^, and other objects. For more details see the review article^14^.

In this work, we propose a new mechanism able to generate lateral forces not subjected to the restriction of local motion imposed by surface corrugations. The force is induced by fluctuations of the electromagnetic field and not by quantum (zero field) fluctuations. If the absorbing medium is anisotropic and the anisotropy axis is tilted with respect to the slab surface, absorption of the TM-polarized wave incident on the slab is different for positive and negative incident angles, although the reflection be the same^21, 22^. The net force induced moves the particle in a direction parallel to the surface.

**Figure 1:**
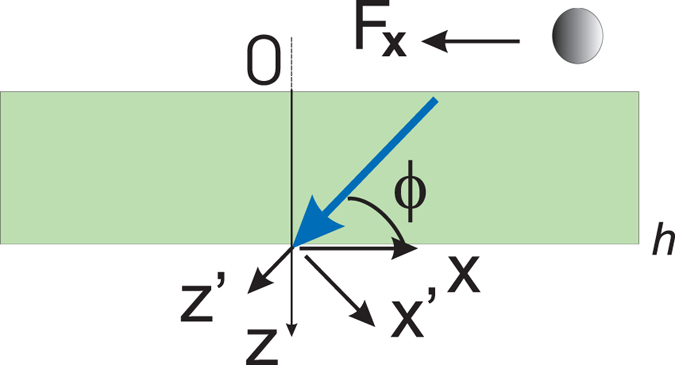
The anisotropic slab and the small particle affected by the lateral force. The anisotropy axis, indicated by a thick blue arrow, forms an angle *ϕ* with the slab interface. Its thickness is *h*. Reference frames (*x*, *y*, *z*) and (*x*′, *y*′, *z*′) are associated with the slab interface and the anisotropy axis, respectively. The black arrow shows the direction of motion of the particle due to the action of the *x*-component of the radiative forces **F**_*x*_.

To calculate the lateral force, we solved the boundary-value problem for electromagnetic waves, excited by point-like fluctuating currents within a finite-thickness slab of an anisotropic medium (see Fig. 1). The correlations of the current are given by fluctuating electrodynamics^5^. Due to the homogeneity of the considered geometry in the *x* and *y* directions, the electric and magnetic fields and the current can be represented by means of their corresponding Fourier transforms **E**(*ω*, *k*_*x*_, *k*_*y*_), **H**(*ω*, *k*_*x*_, *k*_*y*_), and **j**(*ω*, *k*_*x*_, *k*_*y*_). To find a fully accurate solution of the electromagnetic fields is a difficult task because the fields in the considered geometry cannot be decomposed into TM and TE waves. To show the existence of a lateral force, however, it is enough to consider TM waves propagating along the slab in the anisotropy plane, assuming *k*_*y*_ = 0.

The article is organized as follows. In section II, we solve the boundary value problem for electromagnetic waves propagating in the anisotropic slab and calculate the normal and lateral components of the Poynting vector. In Section III, we obtain the eigenwaves. Section IV is devoted to the calculation of the radiative force on a dipole particle. Finally, in Section V, we present our results for the lateral force.

## Normal and lateral components of the Poynting vector

In the coordinate system (*x*′, *y*′, *z*′) (see Fig. 1) the relative permittivity tensor has the diagonal form

1 $$\bar{\bar{\epsilon }}^{\prime} ={\epsilon }_{\parallel }{{\bf{z}}}_{0}^{^{\prime} }{{\bf{z}}}_{0}^{^{\prime} }+{\epsilon }_{t}({{\bf{x}}}_{0}^{^{\prime} }{{\bf{x}}}_{0}^{^{\prime} }+{{\bf{y}}{\boldsymbol{^{\prime} }}}_{0}{{\bf{y}}{\boldsymbol{^{\prime} }}}_{0}\mathrm{)}.$$

where the subscript 0 denotes unit vector. The components of the permittivity tensor in the reference frame *x*, *y*, *z*, associated with the slab interface $\bar{\bar{\epsilon }}$ are the following^21^:

2 $$\begin{array}{l}{\epsilon }_{xz}={\epsilon }_{zx}=({\epsilon }_{\parallel }-{\epsilon }_{t})\cos \,\varphi \,\sin \,\varphi \\ {\epsilon }_{xx}={\epsilon }_{\parallel }{\sin }^{2}\varphi +{\varepsilon }_{t}{\cos }^{2}\varphi \\ {\epsilon }_{zz}={\epsilon }_{\parallel }{\cos }^{2}\varphi +{\varepsilon }_{t}{\sin }^{2}\varphi .\end{array}$$

If the anisotropy axis is tilted with respect to the slab interfaces, the Maxwell equations can be split up into TM and TE subsystems, provided that the wave vector lies in the anisotropy axis plane or is orthogonal to it. We will restrict our analysis to TW waves.

The propagation constants of those waves, travelling along the *z*-direction for a fixed *k*_*x*_ are given by^21^

3 $${k}_{z}^{\mathrm{(1,2)}}=\frac{-{k}_{x}{\epsilon }_{xz}\pm \sqrt{({\epsilon }_{xz}^{2}-{\epsilon }_{xx}{\epsilon }_{zz})({k}_{x}^{2}-{k}_{0}^{2}{\epsilon }_{zz})}}{{\epsilon }_{zz}}.$$

where *k*_0_ is the wavenumber in vacuum. The transverse wave impedances *Z*_1,2_, connecting tangential field components, reads as^21^

4 $${Z}_{1,2}=\frac{{E}_{x}}{{H}_{y}}=\pm \frac{\eta }{{k}_{0}}\frac{\sqrt{{k}_{x}^{2}-{k}_{0}^{2}{\epsilon }_{zz}}}{\sqrt{{\epsilon }_{xz}^{2}-{\epsilon }_{xx}{\epsilon }_{zz}}},$$

where *η* = 120*π* Ohm is the wave impedance of vacuum.

For the tangential field components X(*z*) = (*E*_*x*_(*z*), *H*_*y*_(*z*)), excited by the fluctuating currents *j*_*x*_(*z*), *j*_*z*_(*z*) located within the absorptive layer 0 < *z* < *h* (see Fig. 1), the Maxwell equations reduce to the system of two ordinary differential equations:

5 $$\frac{d}{dz}{\rm{X}}(z)=[{\rm{A}}]{\rm{X}}(z)+{\rm{F}}(z)$$

where the matrix elements of [A] are given by

6 $$\begin{array}{ll}{A}_{11}=-i\frac{{k}_{x}{\epsilon }_{xz}}{{\epsilon }_{zz}}, & {A}_{12}=i\eta {k}_{0}(1-\frac{{k}_{x}^{2}}{{k}_{0}^{2}{\epsilon }_{zz}})\\ {A}_{21}=i\frac{{k}_{0}}{\eta }({\epsilon }_{xx}-\frac{{\epsilon }_{xz}^{2}}{{\epsilon }_{zz}}), & {A}_{22}=-i{k}_{x}\frac{{\epsilon }_{xz}}{{\epsilon }_{zz}}\end{array}$$

and the components of the vector F(*z*) = (*F*_1_(*z*), *F*_2_(*z*)) are

7 $$\begin{array}{rcl}{F}_{1}(z) & = & \eta \,\frac{{k}_{x}}{{k}_{0}{\epsilon }_{zz}}{j}_{z}(z)=a{j}_{z}(z),\\ {F}_{2}(z) & = & \frac{{\epsilon }_{xz}}{{\epsilon }_{zz}}{j}_{z}(z)-{j}_{x}(z)=b{j}_{z}(z)-{j}_{x}(z),\end{array}$$

The elementary bulk current source has the form: **j**(*z*) = **j**_0_(*z*′)*δ*(*z* − *z*′).

The solution of Eq. (5) for points 0 < *z* < *h* is^23^:

8 $${\rm{X}}(z)={e}^{[{\rm{A}}]z}{\rm{X}}\mathrm{(0)}+{\int }_{0}^{z}{e}^{[{\rm{A}}](z-\tau )}{\rm{F}}(\tau )d\tau ,$$

with [M(*z*)] = *e* ^[A]*z*^ the transfer matrix. Expressions for the two-by-two matrix components, for the case in which the wave impedances and vector components are different for waves propagating in opposite directions, are given in refs 21, 22. For the considered case, where *Z*_2_ = −*Z*_1_ = *Z*, those expressions reduce to

9 $$\begin{array}{ll}{M}_{11}(z)=\frac{1}{2}[{e}^{i{k}_{z1}z}+{e}^{i{k}_{z2}z}] & {M}_{12}(z)=\frac{Z}{2}[{e}^{i{k}_{z1}z}-{e}^{i{k}_{z2}z}]\\ {M}_{21}(z)=\frac{1}{Z}[{e}^{i{k}_{z1}z}-{e}^{i{k}_{z2}z}] & {M}_{22}(z)={M}_{11}(z\mathrm{)}.\end{array}$$

The boundary conditions are: *X*_2_(0) = *X*_1_(0)/*Z*_0_, *X*_2_(*h*) = −*X*_1_/*Z*_0_, where ${Z}_{0}=\eta \sqrt{({k}_{0}^{2}-{k}_{x}^{2})}/{k}_{0}$ is the *transverse* wave impedance in vacuum. We can then express the tangential field components at the interface *x* = 0, created by a current located at *z*′ in the form

10 $${X}_{1}\mathrm{(0,}z^{\prime} )=\frac{1}{{\rm{\Delta }}}{\int }_{0}^{h}[U(\tau ){j}_{x0}(z^{\prime} )+V(\tau ){j}_{z0}(z^{\prime} )]\delta (\tau -z^{\prime} )\,d\tau =\frac{1}{{\rm{\Delta }}}[U(z^{\prime} ){j}_{x0}(z^{\prime} )+V(z^{\prime} ){j}_{z0}(z^{\prime} )],$$

where

11 $$\begin{array}{rcl}{\rm{\Delta }} & = & {M}_{11}(h)+{M}_{22}(h)-{M}_{12}(h)/{Z}_{0}-{M}_{21}(h){Z}_{0},\\ U(\tau ) & = & {M}_{12}(h-\tau )-{Z}_{0}{M}_{22}(h-\tau ),\\ V(\tau ) & = & a[{Z}_{0}{M}_{21}(h-\tau )-{M}_{11}(h-\tau )]+b[{Z}_{0}{M}_{22}(h-\tau )-{M}_{12}(h-\tau \mathrm{)]}.\end{array}$$

The Fourier components of the electric and magnetic fields out of the layer are given by

12 $${E}_{x}({k}_{x},z,z^{\prime} )=\{\begin{array}{l}{X}_{1}\mathrm{(0},z^{\prime} ){e}^{-i{k}_{z0}z},z < \mathrm{0,}\\ {X}_{1}(h,z^{\prime} ){e}^{i{k}_{z0}(z-h)},z > h,\end{array}$$

where ${k}_{z0}=\sqrt{{k}_{0}^{2}-{k}_{x}^{2}}$ and *H*_*y*_(*k*_*x*_, *z*, *z*′) = *E*_*x*_(*k*_*x*_, *z*, *z*′)/*Z*_0_. For evanescent waves, |*k*_*x*_| > *k*_0_, we have to take *k*_*z*0_ = −*i*|*k*_*z*0_|, if *z* < 0 and *k*_*z*0_ = *i*|*k*_*z*0_|, if *z* > *h*.

The average values of the fluctuating currents vanish, only their correlations contribute to the energy flux. These correlations are given through the fluctuation-dissipation theorem^24^ The ensemble-averaged Poynting vector in the plane *z* = 0, for the *k*_*x*_ mode, induced by fluctuating currents located within the slab, 0 < *z*′, *z*″ < *h*, reads

13 $$\langle {S}_{z}(\omega ,{k}_{x})\rangle =\frac{1}{2}{\int }_{0}^{h}{\int }_{0}^{h}\langle {E}_{x}({k}_{x},z^{\prime} ){H}_{y}^{\ast }({k}_{x},z^{\prime\prime} )\rangle dz^{\prime} dz^{\prime\prime} ,$$

where the correlation $\langle {E}_{x}({k}_{x},z^{\prime} ){H}_{y}^{\ast }({k}_{x},z^{\prime\prime} )\rangle$ is obtained by using the fluctuation-dissipation theorem^24^

14 $$\langle {j}_{m}({\bf{r}},\omega ){j}_{n}^{\ast }({\bf{r}}{\boldsymbol{^{\prime} }},\omega ^{\prime} )\rangle =\frac{4}{\pi }\omega {\epsilon }_{0}{\epsilon }_{mn}^{^{\prime\prime} }(\omega )\delta ({\bf{r}}-{\bf{r}}{\boldsymbol{^{\prime} }})\delta (\omega -\omega ^{\prime} ){\rm{\Theta }}(\omega ,T),$$

with $$\overrightarrow{r}=(x,z)$$ and

15 $${\rm{\Theta }}(\omega ,T)=\frac{\hslash \omega }{{e}^{\hslash \omega /({k}_{B}T)}-1}$$

the Planck’s oscillator energy. Contribution from vacuum fluctuations is omitted since thermal fluctuations only contribute to the lateral forces. In Eq. (14), ${\epsilon }_{mn}^{^{\prime\prime} }\equiv {\rm{Im}}({\epsilon }_{mn})$, ${\epsilon }_{0}$ is the permittivity of vacuum, *ħ* the reduced Planck constant, *T* the temperature, and *k*_*B*_ the Boltzmann constant.

Using the fluctuation-dissipation theorem (14), we then obtain

16 $$\langle {S}_{z}(\omega ,{k}_{x})\rangle =\frac{4\omega {\epsilon }_{0}{\rm{\Theta }}(\omega ,T)}{2\pi {|{\rm{\Delta }}|}^{2}{Z}_{0}^{\ast }}[{D}_{1}{\epsilon }_{xx}^{^{\prime\prime} }+{D}_{2}{\epsilon }_{zz}^{^{\prime\prime} }+{D}_{3}{\epsilon }_{xz}^{^{\prime\prime} }],$$

where we have defined the coefficients

17 $$\begin{array}{rcl}{D}_{1} & = & {\int }_{0}^{h}U(\tau ){U}^{\ast }(\tau )d\tau ,\\ {D}_{2} & = & {\int }_{0}^{h}V(\tau ){V}^{\ast }(\tau )d\tau ,\\ {D}_{3} & = & {\int }_{0}^{h}U(\tau ){V}^{\ast }(\tau )d\tau +c.c,\end{array}$$

The expressions for these coefficients are found to be

18 $$\begin{array}{rcl}{D}_{1} & = & \frac{1}{4}\{\frac{i{|Z-{Z}_{0}|}^{2}}{({k}_{z1}-{k}_{z1}^{\ast })}\mathrm{(1}-{e}^{i({k}_{z1}-{k}_{z1}^{\ast })h})+\frac{i{|Z+{Z}_{0}|}^{2}}{({k}_{z2}-{k}_{z2}^{\ast })}\mathrm{(1}-{e}^{i({k}_{z2}-{k}_{z2}^{\ast })h})\\  &  & -[\frac{i(Z-{Z}_{0})({Z}^{\ast }+{Z}_{0}^{\ast })}{({k}_{z1}-{k}_{z2}^{\ast })}\mathrm{(1}-{e}^{i({k}_{z1}-{k}_{z2}^{\ast })h})+c.c.]\}.\end{array}$$

19 $$\begin{array}{rcl}{D}_{2} & = & \frac{{|a|}^{2}}{4}\{\frac{i{|{Z}_{0}/Z-1|}^{2}}{({k}_{z1}-{k}_{z1}^{\ast })}(1-{e}^{i({k}_{z1}-{k}_{z1}^{\ast })h})+\frac{i{|{Z}_{0}/Z+1|}^{2}}{({k}_{z2}-{k}_{z2}^{\ast })}(1-{e}^{i({k}_{z2}-{k}_{z2}^{\ast })h})\\  &  & +-[[\frac{i({Z}_{0}/Z-1)({Z}_{0}^{\ast }/{Z}^{\ast }+1)}{({k}_{z1}-{k}_{z2}^{\ast })}(1-{e}^{i({k}_{z1}-{k}_{z2}^{\ast })h})+c.c.]\}\\  &  & +\,\frac{{|b|}^{2}}{4}\{\frac{i{|{Z}_{0}-Z|}^{2}}{({k}_{z1}-{k}_{z1}^{\ast })}(1-{e}^{i({k}_{z1}-{k}_{z1}^{\ast })h})+\frac{i{|{Z}_{0}+Z|}^{2}}{({k}_{z2}-{k}_{z2}^{\ast })}(1-{e}^{i({k}_{z2}-{k}_{z2}^{\ast })h})\\  &  & +\,[\frac{i({Z}_{0}-Z)({Z}_{0}^{\ast }+{Z}^{\ast })}{({k}_{z1}-{k}_{z2}^{\ast })}(1-{e}^{i({k}_{z1}-{k}_{z2}^{\ast })h})+c.c.]\}\\  &  & +\,\{\frac{a{b}^{\ast }}{4Z}[\frac{i{|{Z}_{0}-Z|}^{2}}{{k}_{z1}-{k}_{z1}^{\ast }}(1-{e}^{i({k}_{z1}-{k}_{z1}^{\ast })h})-\frac{i{|{Z}_{0}+Z|}^{2}}{{k}_{z2}-{k}_{z2}^{\ast }}(1-{e}^{i({k}_{z2}-{k}_{z2}^{\ast })h})\\  &  & +\frac{i({Z}_{0}-Z)({Z}_{0}^{\ast }+{Z}^{\ast })}{{k}_{z1}-{k}_{z2}^{\ast }}(1-{e}^{i({k}_{z1}-{k}_{z2}^{\ast })h})\\  &  & -\frac{i({Z}_{0}+Z)({Z}_{0}^{\ast }-{Z}^{\ast })}{{k}_{z2}-{k}_{z1}^{\ast }}(1-{e}^{i({k}_{z2}-{k}_{z1}^{\ast })h})]+c.c.\}.\end{array}$$

20 $$\begin{array}{rcl}{D}_{3} & = & \frac{1}{4}[\frac{i{C}_{1}(Z-{Z}_{0})}{{k}_{z1}-{k}_{z1}^{\ast }}(1-{e}^{i({k}_{z1}-{k}_{z1}^{\ast })h})+\frac{i{C}_{2}(Z+{Z}_{0})}{{k}_{z2}-{k}_{z2}^{\ast }}(1-{e}^{i({k}_{z2}-{k}_{z2}^{\ast })h})\\  &  & -\frac{i{C}_{2}(Z-{Z}_{0})}{{k}_{z1}-{k}_{z2}^{\ast }}(1-{e}^{i({k}_{z1}-{k}_{z2}^{\ast })h})-\frac{i{C}_{1}(Z+{Z}_{0})}{{k}_{z2}-{k}_{z1}^{\ast }}(1-{e}^{i({k}_{z2}-{k}_{z1}^{\ast })h})],\end{array}$$

with

21 $$\begin{array}{rcl}{C}_{1} & = & {a}^{\ast }{Z}_{0}^{\ast }/Z-{a}^{\ast }+{b}^{\ast }{Z}_{0}^{\ast }-{b}^{\ast }{Z}^{\ast },\\ {C}_{2} & = & {a}^{\ast }{Z}_{0}^{\ast }/Z+{a}^{\ast }-{b}^{\ast }{Z}_{0}^{\ast }-{b}^{\ast }{Z}^{\ast }.\end{array}$$

If the anisotropy axis is parallel or orthogonal to the interface, then 〈*S*_*z*_(*ω*, *k*_*x*_)〉 = 〈*S*_*z*_(*ω*, −*k*_*x*_)〉. Otherwise, that average becomes asymmetric with respect to the normal to the slab interface, as occurs in absorption^21, 22^. This value is real if |*k*_*x*_| < *k*_0_ and imaginary if |*k*_*x*_| > *k*_0_. Actually, the real part of 〈*S*_*z*_(*ω*, *k*_*x*_)〉 determines the far-field thermal emission at the angle *θ* = *arcsin k*_*x*_/*k*_0_.

Outside the slab, the lateral time-averaged component of the Poynting vector is given by

22 $$\langle {S}_{x}(\omega ,{k}_{x},z)\rangle =-\frac{1}{2}{E}_{z}{H}_{y}^{\ast }=\frac{{k}_{x}}{{k}_{z0}}\langle {S}_{z}(\omega ,{k}_{x})\rangle f(z),$$

where *f*(*z*) = 1, if |*k*_*x*_| < *k*_0_, and $f(z)={e}^{2|{k}_{z0}|z}$ (*z* < 0), if |*k*_*x*_| > *k*_0_. Unlike 〈*S*_*z*_(*ω*, *k*_*x*_)〉, this value is real for all *k*_*x*_, i.e. both for propagating and evanescent waves. The contribution of all *k*_*x*_-modes to the *x*-component of the Poynting vector outside the slab is given as:

23 $$\langle {S}_{x}(\omega )\rangle =\frac{1}{2\pi }{\int }_{-\infty }^{\infty }\langle {S}_{x}(\omega ,{k}_{x},z)\rangle d{k}_{x}.$$

Despite the fact that the lateral component of the Poynting vector is real for all *k*_*x*_, propagating waves only give contribution to the overall value (23), hence 〈*S*_*x*_(*ω*, *k*_*x*_, *z*)〉 ≠ 〈*S*_*x*_(*ω*, −*k*_*x*_, *z*)〉, if |*k*_*x*_| < *k*_0_ and 〈*S*_*x*_(*ω*, *k*_*x*_, *z*)〉 = 〈*S*_*x*_(*ω*, −*k*_*x*_, *z*)〉, if |*k*_*x*_| ≥ *k*_0_. These results come from the fact that the field correlators outside a finite-thickness slab of an absorbing medium are expressed via transmission and reflection coefficients for the propagating waves spectrum and via reflection coefficients only for evanescent waves (see, for example^25^, Eq. (120), and ref. 26, Eq. (103)). For the slab made of an anisotropic material with a tilted anisotropy axis the reflection coefficients are symmetric with respect to ± *k*_*x*_ for any *k*_*x*_, whereas the transmission coefficients are asymmetric.

Since 〈*S*_*x*_(*ω*, *k*_*x*_, *z*)〉 ≠ 〈*S*_*x*_(*ω*, −*k*_*x*_, *z*)〉 (|*k*_*x*_| < *k*_0_), one can expect the appearance of radiative forces dragging a particle placed nearby the slab along its surface.

When |*k*_*x*_| < *k*_0_, expression (13) gives us the thermal power radiating from the slab in the anisotropy axis (*x*, *z*) plane, i.e. for *k*_*y*_ = 0. The total energy flux density in the *x*-direction, produced by electromagnetic fluctuations, is given by

24 $${\langle {S}_{x}(z)\rangle }^{{\rm{tot}}}={\int }_{0}^{\infty }\frac{d\omega }{{\mathrm{(2}\pi )}^{3}}\int {\int }_{-{k}_{0}}^{{k}_{0}}\langle {S}_{x}(\omega ,{k}_{x},{k}_{y})\rangle d{k}_{x}d{k}_{y}.$$

An exact value of this quantity for nonzero values of *k*_*x*_ and *k*_*y*_ is difficult to obtain since the fluctuating fields in the slab are carried by hybrid waves whose solution is more difficult to obtain than that for TM waves.

The calculation of the total energy flux in the *x*-direction is based on the following consideration. If the anisotropy axis is orthogonal to the slab interfaces or if the medium is isotropic, due to azimuthal symmetry, one can replace *dk*_*x*_ *dk*_*y*_ by 2*πk*_*x*_ *dk*_*x*_, therefore

25 $${\int }_{-{k}_{0}}^{{k}_{0}}{\int }_{-{k}_{0}}^{{k}_{0}}\langle {S}_{z}(\omega ,{k}_{x},{k}_{y})\rangle \,d{k}_{x}\,d{k}_{y}=2\pi {\int }_{0}^{{k}_{0}}\langle {S}_{z}(\omega ,q)\rangle q\,dq$$

and ${\langle {S}_{x}(z)\rangle }^{{\rm{tot}}}=\langle {S}_{x}^{s}(z)\rangle +\langle {S}_{x}^{p}(z)\rangle \equiv 0$. Let us consider separately the cases in which the wave vector is either parallel or orthogonal to the plane normal to the slab surface, containing the anisotropy axis. In both cases, waves in an anisotropic slab can be split up into *p*-polarized and *s*-polarized waves. Obviously, for the *s*-polarized waves the asymmetry never takes place and $\langle {S}_{x}^{p}(z)\rangle \equiv 0$ for both cases, *k*_*x*_ ≠ 0, *k*_*y*_ = 0 and *k*_*x*_ = 0, *k*_*y*_ ≠ 0. For *p*-polarized waves, the asymmetry is absent if *k*_*x*_ = 0, at any *k*_*y*_ and it is maximal if *k*_*x*_ ≠ 0, *k*_*y*_ = 0. Thus, the integral over waves, propagating in the *y*-direction, i.e. *k*_*x*_ = 0, gives zero contribution to $\langle {S}_{c}^{p}(z)\rangle$. A good approximation to the total lateral energy flux density is then given by

26 $${\langle {S}_{x}^{{\rm{appr}}}(z)\rangle }^{{\rm{tot}}}\approx \frac{1}{4\pi }{\int }_{0}^{\infty }\,d\omega {\int }_{-{k}_{0}}^{{k}_{0}}\langle {S}_{x}^{p}(\omega ,q)\rangle q\,dq.$$

Asymmetry with respect to ±*k*_*x*_ takes place for any absorbing anisotropic material but it becomes particularly important for media characterized by hyperbolic dispersion, for the so-called *hyperbolic materials*, whose diagonal components of the permittivity tensor have different signs. To illustrate the lateral drag effect, we will consider the orthorhombic modification of boron nitride which exhibits hyperbolic dispersion in certain frequency ranges^27, 28^.

## The Lorentzian model and the eigenwaves in a boron nitride medium

The components of the permittivity tensor are given by the Lorentz model^27, 28^:

27 $${\epsilon }_{\parallel ,\perp }={\epsilon }_{\parallel ,\perp }^{\infty }+\frac{{U}_{\parallel ,\perp }{({\omega }_{\parallel ,\perp }^{\tau })}^{2}}{{({\omega }_{\parallel ,\perp }^{\tau })}^{2}-{\omega }^{2}-i\omega {{\rm{\Gamma }}}_{\parallel ,\perp }},$$

where ${\omega }_{\parallel ,\perp }^{\tau }$ and ${U}_{\parallel ,\perp }$ are, respectively, the transverse phonon frequency and the oscillator strength of the lattice vibration for the parallel and perpendicular polarizations, and ${{\rm{\Gamma }}}_{\parallel ,\perp }$ is the damping constant. The constants ${\epsilon }_{\parallel ,\perp }^{\infty }$ are the components of the permittivity tensor at frequencies *ω* that greatly exceed the phonon resonance frequency ${\omega }_{\parallel ,\perp }^{\tau }$. The values of the parameters of (27) used are: ${\epsilon }_{\parallel }^{\infty }=2.7$, ${U}_{\parallel }=0.48$, ${\omega }_{\parallel }^{\tau }=1.435\times {10}^{14}$ rad/s, ${{\rm{\Gamma }}}_{\parallel }=8.175\times {10}^{11}$ rad/s, ${\epsilon }_{\perp }^{\infty }=5.2$, *U*_⊥_ = 2, ${\omega }_{\perp }^{\tau }=2.588\times {10}^{14}$ rad/s, Γ_⊥_ = 1.29 × 10^12^ rad/s. For this parameters, the Lorentzian resonances of ${\epsilon }_{\parallel }$ and ${\epsilon }_{\perp }$, take place at frequencies ≈22.8 THz and ≈41.2 THz, respectively.

The propagation constants and the transverse wave impedance of the TM waves, traveling along the *z*-direction under fixed *k*_*x*_, are given by Eqs (3) and (4), respectively. In order to correctly identify the waves propagating in the positive and negative directions of the *z*-axis, we have to analyze the imaginary parts of ${k}_{z}^{\mathrm{(1,2)}}$, according to the causality principle. Let us define the normal wave vector component for the downward wave (propagating in the positive *z*-direction) as *k*_1_ and for the upward wave as *k*_2_. One then has: Im (*k*_1_) > 0 and Im (*k*_2_) < 0. The parallel ${\epsilon }_{\parallel }$ and perpendicular ${\epsilon }_{\perp }$ components of the permittivity tensor of orthorhombic boron nitride, taken for illustration of the predicted effect, exhibit the Lorentzian resonances at frequencies ≈22.8 THz and ≈41.2 THz, respectively. It is expected, that *k*_1_ and *k*_2_ exhibit a resonant behavior nearby these frequencies.

**Figure 2:**
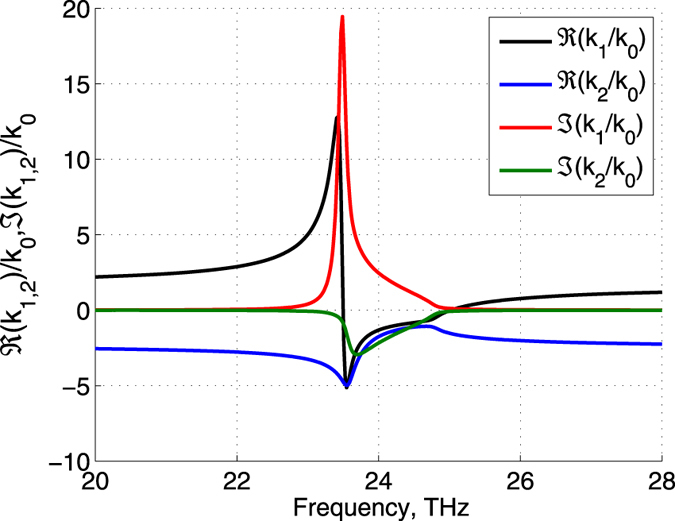
Real and imaginary parts of *k*_1_ and *k*_2_, normalized to the wavenumber in vacuum *k*_0_, versus frequency in the vicinity of the ${\epsilon }_{\parallel }$- resonance.

**Figure 3:**
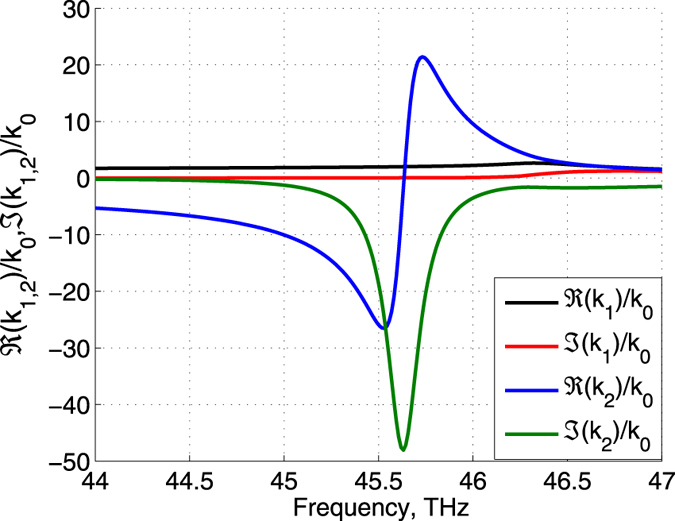
Real and imaginary parts of *k*_1_ and *k*_2_, normalized to the wavenumber in vacuum *k*_0_, versus frequency in the vicinity of the ${\epsilon }_{\perp }$- resonance.

Figure 2 illustrates the frequency dependencies of the real and imaginary parts of the normal components *k*_1_, *k*_2_ of wave vectors. One can see that Re(*k*_1_) changes the sign in the vicinity of the resonance, so the downward propagating wave becomes the forward wave at low frequencies and the backward wave in the frequency range from ≈23.6 THz to ≈25 THz. The upward wave remains the forward one within the considered range. Figure 3 shows similar dependencies on the frequency range around the ${\epsilon }_{\perp }$ resonance. Unlike the previous case, here the upward wave changes the sign of dispersion.

**Figure 4:**
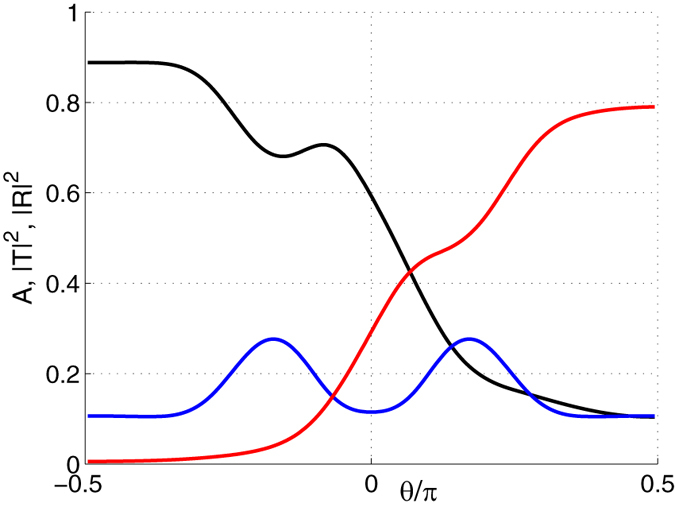
Absorption *A* (black), transmission |*T*|^2^ (red) and reflection |*R*|^2^(blue) of the plane wave incident onto the slab of orthorhombic boron nitride versus the incidence angle *θ* [rad]. The thickness of the layer *h* is 1.5 *μ*m and the tilt angle *ϕ* = 40°.

The dependence of the transmission, |*T*|^2^, reflection, |*R*|^2^, and absorption *A* = 1 − |*T*|^2^ − |*R*|^2^ on the incidence angle, calculated at 45 THz which is close to the Lorentzian resonance for ${\epsilon }_{\parallel }$, is shown in Fig. 4. The displayed dependence of the absorption versus the incidence angle is the signature of the asymmetry of thermal emission and radiative forces with respect to *k*_*x*_.

## Radiative forces on a dipole particle

To evaluate the effect of the lateral forces, we will consider a small particle moving under the influence of fluctuating electromagnetic fields. The dipolar force acting on the particle can be written as^29^

28 $$\langle {\bf{F}}\rangle =\frac{1}{4}{\rm{Re}}\{\alpha \}\nabla {|{\bf{E}}|}^{2}+\sigma \frac{1}{2}{\rm{Re}}\{\frac{1}{c}{\bf{E}}\times {{\bf{H}}}^{\ast }\}+\sigma \frac{1}{2}{\rm{Re}}\{i\frac{{\epsilon }_{0}}{{k}_{0}}({\bf{E}}\cdot \nabla ){{\bf{E}}}^{\ast }\}$$

where *α* is the polarizability of the particle given by

29 $$\alpha =\frac{{\alpha }_{0}}{1-i{\alpha }_{0}{k}_{0}^{3}\mathrm{/(6}\pi {\epsilon }_{0})},\quad {\alpha }_{0}=4\pi {\epsilon }_{0}{r}^{3}\frac{\epsilon -1}{\epsilon +2},$$

with *r* and $\epsilon$ its radius and permittivity, respectively, and *σ* = *k*_0_Im{*α*}/${\epsilon }_{0}$. The origin of the force is the presence of thermal fluctuations in the anisotropic slab which give rise to thermal emission from the slab into the far zone. Finite thickness of the slab and tilted anisotropy axis are conditions necessary to generate the force. Zero-field fluctuations do not give a contribution to the force since the energy flows incoming from the left and the right sides balance each other out.

The first term in (28), related to the gradient forces, causes attraction of the particle toward the slab interface due to the *z*-dependence of fields through ${e}^{|{k}_{z0}|z}$, for *z* < 0 (van-der-Waals forces^1^). The explicit expression for ∇|**E**|^2^ is

30 $$\nabla {|{\bf{E}}(\omega ,{k}_{x},z)|}^{2}=\frac{\partial }{\partial z}f(z)[\langle {E}_{x}{E}_{x}^{\ast }\rangle +\langle {E}_{z}{E}_{z}^{\ast }\rangle ]=2\langle {S}_{z}(\omega ,{k}_{x})\rangle \eta f(z)\frac{2{k}_{x}^{2}-{k}_{0}^{2}}{{k}_{0}},$$

Only the evanescent waves (|*k*_*x*_| > *k*_0_) contribute to this force.

In the second contribution, the *x*- and *z*-component of the Poynting vector exert pulling forces along the corresponding directions. At small |*z*|, the attractive gradient force is dominant, whereas at larger |*z*| the dominant force is the repulsive force proportional to the *z* component of the Poynting vector.

Due to the fact that

31 $$\begin{array}{rcl}{\langle ({\bf{E}}\cdot \nabla ){{\bf{E}}}^{\ast }\rangle }_{x} & = & {E}_{x}\frac{\partial }{\partial x}{E}_{x}^{\ast }+{E}_{z}\frac{\partial }{\partial z}{E}_{x}^{\ast }=0,\\ {\langle ({\bf{E}}\cdot \nabla ){{\bf{E}}}^{\ast }\rangle }_{z} & = & {E}_{x}\frac{\partial }{\partial x}{E}_{z}^{\ast }+{E}_{z}\frac{\partial }{\partial z}{E}_{z}^{\ast }=0,\end{array}$$

the third term in (28) does not contribute to the radiative forces. The *x*-component of the Poynting vector in (28) is given by Eq. (26) and the *z*-component can be found by replacing $\langle {S}_{x}^{p}(\omega ,q)\rangle$ by $\langle {S}_{z}^{p}(\omega ,q)\rangle =-({k}_{z0}/{k}_{x})\langle {S}_{x}^{p}(\omega ,q)\rangle$.

Note, that since the TM (p)-polarized waves only contribute to the lateral Poynting vector at *k*_*y*_ = 0 and in our approximation we have not taken into account the hybrid nature of waves in the anisotropic slab at *k*_*y*_ ≠ 0, we can consider contributions of the p-polarized waves only to the *x*-directed forces.

As an example of particle experiencing a lateral drag force, we consider a spherical nanoparticle, made of doped silicon, whose complex permittivity ${\epsilon }_{s}$ in the infrared and far infrared ranges can be calculated by using the Masetti model^30^. This model considers that the permittivity of heavily doped silicon is given by the Drude formula^30, 31^:

32 $$\epsilon (\omega )={\epsilon }_{\infty }-\frac{{\omega }_{p}^{2}}{\omega (\omega -j\gamma )}$$

where ${\epsilon }_{\infty }$ ≈ 11.6 is the high-frequency limit value of the permittivity^32^, and *γ* is the scattering rate which depends on the mobility of the carriers. The plasma frequency and scattering rate are expressed as ${\omega }_{p}=\sqrt{N{e}^{2}/({m}^{\ast }{\epsilon }_{0})}$ and *γ* = *e*/(*m* ^*^ *μ*), respectively, where *e* is the electron charge, *N* is the carrier concentration, *m* ^*^ is the carrier effective mass, and *μ* is the mobility. For *n*-type heavily doped Si, the mobility expression is given as^31^

33 $$\mu ={\mu }_{1}+\frac{{\mu }_{\max }-{\mu }_{1}}{1+{(N/{C}_{r})}^{\alpha }}-\frac{{\mu }_{2}}{1+{({C}_{s}/N)}^{\beta }}.$$

**Figure 5:**
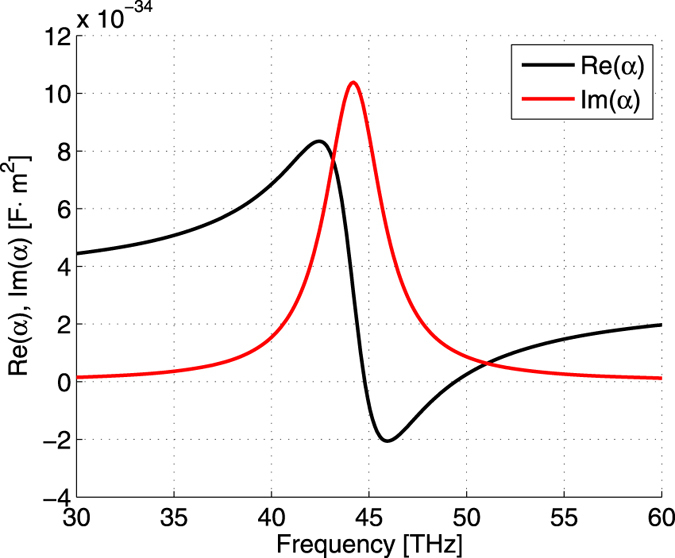
Real and imaginary parts of the polarizability *α* [F · m^2^] of the nanoparticle, made of doped silicon with concentration of carriers 9 × 10^19^ cm^−3^.

Here *m* ^*^ = 0.27*m*_0_, where *m*_0_ is the electron mass, *μ*_1_ = 68.5 cm^2^/V s, *μ*_max_ = 1414 cm^2^/V s, *μ*_2_ = 56.1 cm ^2^/V s, *C*_*r*_ = 9.2 × 10^17^ cm^−3^, *C*_*s*_ = 3.42 × 10^20^ cm^−3^, *α* = 0.711 and *β* = 1.98. For these parameters, *ω*_*p*_ = 1.084 × 10^15^ rad/s and *γ* = 8.586 × 10^13^ rad/s. The concentration of carriers is 9 × 10^19^ cm^−3^ and its radius 15 nm. For these values of the parameters the particle exhibits a dipole resonance within the same frequency range as for the perpendicular component of the permittivity tensor of orthorhombic boron nitride, namely, around 44 THz, see Fig. 5. In the figure, one can see a very strong enhancement of Im (*α*), proportional to the lateral force, within the frequency band, providing main contribution to the lateral component of the Poynting vector.

## Results and Discussion

**Figure 6:**
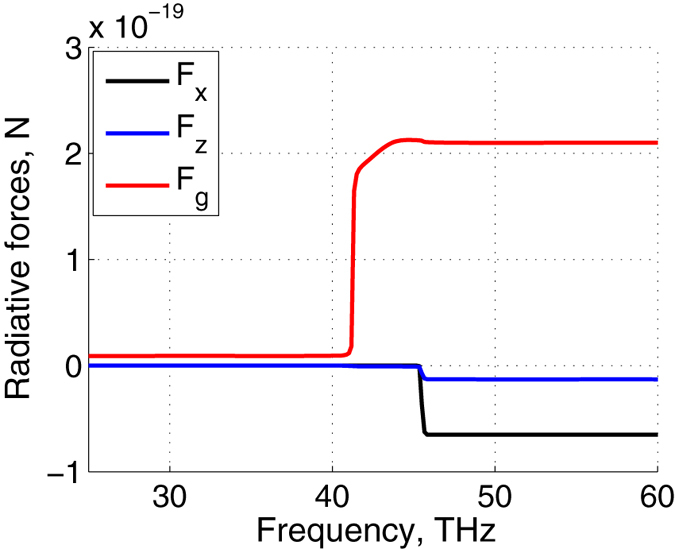
Radiative forces [N] versus frequency for *h* = 400 nm, *T* = 450 K, and *ϕ* = 50°: *F*_*g*_ is the conservative, attractive gradient force, exerted by non-homogeneous fields of evanescent waves (red); *F*_*x*_ (black) and *F*_*z*_ (blue) are the lateral and normal repulsive, non-conservative forces, induced by the corresponding components of the Poynting vector, respectively.

Figure 6 shows the dipolar forces acting on the particle, computed at *z* = 1 *μ*m, and integrated in frequencies. The lower integration limit is 20 THz since below this frequency contributions to all radiative forces are very small. The upper limit corresponds to a current frequency at the abscissa axis. One can see that the main contributions to all forces come from the frequency band 41–45 THz corresponding to the Lorentz resonance for *k*_*z*_. Further integration does not change the results; saturation is observed after 47 THz.

The normal component of the force consists of contributions from the gradient force, *F*_*g*_, and the force, caused by the Poynting vector, *F*_*z*_. One can verify, that at such a distance the van-der-Waals attractive force is comparable with the lateral force. The magnitude of the lateral drag force is even stronger than the repulsive *z*-directed force. The spectral densities of these forces were calculated and pictured within the frequency range 20 THz–60 THz in which the Lorentz model for orthorhombic boron nitride (27) applies, see Fig. 6. Our estimations thus show that the predicted lateral force could be detected experimentally. Visualization of the velocity of the colloidal nanoparticle could be carried out by using the method of particle image velocimetry successfully employed with gold nanoparticles^33^. Under the influence of this lateral force, the acceleration of the nanoparticle is 2.46 m/s^2^. One can expect saturation of the particle speed due to contactless quantum friction. To make that lateral forces are the only ones acting on the particle, one can inhibit the perpendicular Casimir force by placing a non-absorbing layer between the particle and the absorbing layer. In this way, the particle could only move along the lateral direction.

In summary, we have predicted a new effect caused by the fluctuations of the electromagnetic field nearby an absorbing anisotropic slab: the presence of lateral drag forces emerging when the anisotropy axis of the slab is tilted. Electromagnetic fluctuations have been treated within the framework of Rytov’s formalism which constitutes one of the important tools to study fluctuation-induced interactions, and it is applicable at the nanoscale as well^5^. This effect systematically occurs in any absorbing anisotropic media, but it may be especially relevant for materials with a strong anisotropy. To prove the existence of such forces, we have solved the boundary value problem in the TM-waves approximation that ignores the hybrid nature of the waves supported by the slab for the considered anisotropy, if *k*_*y*_ ≠ 0. The presence of these drag forces which can be referred to as “*the driving force from nothing*”^34^ can play an important role in the manipulation of nanoparticles close to a surface.
